# Supplementary material for: Mechanisms of synthetic lethality between BRCA1/2 and 53BP1 deficiencies and DNA polymerase theta targeting
Source: Nat Commun. 2023 Nov 29;14:7834. doi: 10.1038/s41467-023-43677-2 (PMC10687250; doi:10.1038/s41467-023-43677-2)
Supplement: Supplementary file 1 — Supplementary Information [file 41467_2023_43677_MOESM1_ESM.pdf]

Supplementary Information

Supplemental Figure 1.

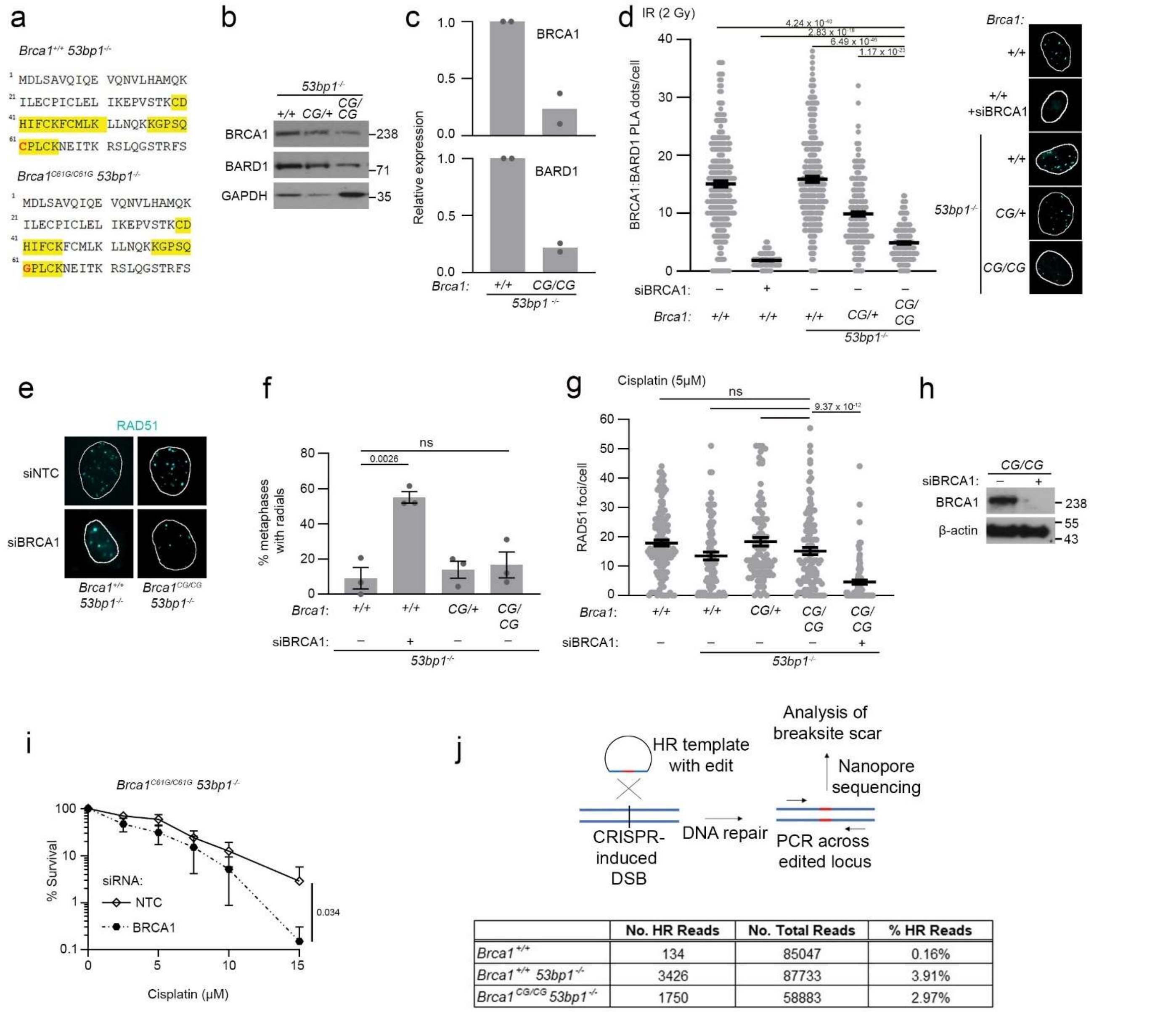

	No. HR Reads	No. Total Reads	% HR Reads
Brca1$^{+/+}$	134	85047	0.16%
Brca1$^{+/+}$ *53bp1*$^{-/-}$	3426	87733	3.91%
Brca1$^{CG/CG}$ *53bp1*$^{-/-}$	1750	58883	2.97%

Supplementary Fig. 1. Brca1$^{C61G/C61G}$ 53bp1$^{-/-}$ cells support HR.

a. BRCA1 N-terminal peptides identified by mass spectrometry, highlighted in yellow, from *Brca1*$^{+/+}$ *53bp1*$^{-/-}$ *and Brca1*$^{C61G/C61G}$ *53bp1*$^{-/-}$ mouse embryonic fibroblasts.

b. Representative western blot analysis of BRCA1, BARD1 protein levels in MEFs. The expression of GAPDH was used as a loading control.

c. BRCA1-BARD1 protein levels in *Brca1*$^{+/+}$ *53bp1*$^{-/-}$ and *Brca1*$^{C61G/C61G}$ *53bp1*$^{-/-}$ cells, relative to GAPDH loading controls and wild-type protein. n=2 biological replicates, data are mean ± SEM.

d. Quantification of proximity-linked ligation assay foci (PLA) between BRCA1 and BARD1 in cells treated with 2 Gy irradiation and with NTC siRNA (−) or siRNA targeting BRCA1 (+) and fixed 2 hours later. n≥200 cells from 5 biological replicates. Bars depict median ± SEM.
e. Representative images of RAD51 foci in *Brca1*$^{+/+}$ *53bp1*$^{-/-}$ *and Brca1*$^{C61G/C61G}$ *53bp1*$^{-/-}$ cells, 3 hours after exposure to 2 Gy irradiation, treated with siNTC or siRNA targeting BRCA1.
f. Percentage of metaphases that show one or more radial chromosomes in MEFs of the genotypes shown, treated with NTC siRNA (−) or siRNA targeting BRCA1 (+). n=3 biological replicates, data are mean ± SEM.
g. Quantification of RAD51 foci following 16 hours 5 µM cisplatin exposure in EdU-positive MEFs of the genotypes shown, treated with NTC siRNA (-) or siRNA targeting BRCA1 (+). n>100 cells from 3 biological replicates. Data are mean ± SEM.
h. Representative western blot showing knockdown of BRCA1 in *Brca1*$^{C61G/C61G}$ *53bp1*$^{-/-}$ cells.
i. Colony survival following 16 hours of treatment with cisplatin, measured in *Brca1*$^{C61G/C61G}$ *53bp1*$^{-/-}$ cells treated with non-targeting control siRNA (NTC) or BRCA1 siRNA transfection, n=3 biological replicates, data are mean ± SEM.
j. Illustration of the HR assay (top). The table below shows the % of reads containing the 4 bp inclusion and the total number of reads.

Statistical analysis in d, f, and g was performed using a two-tailed Student's t-Test, without adjustment for multiple comparisons. Statistical analysis in i was performed using a two-way ANOVA. Source data are provided as a Source Data file.

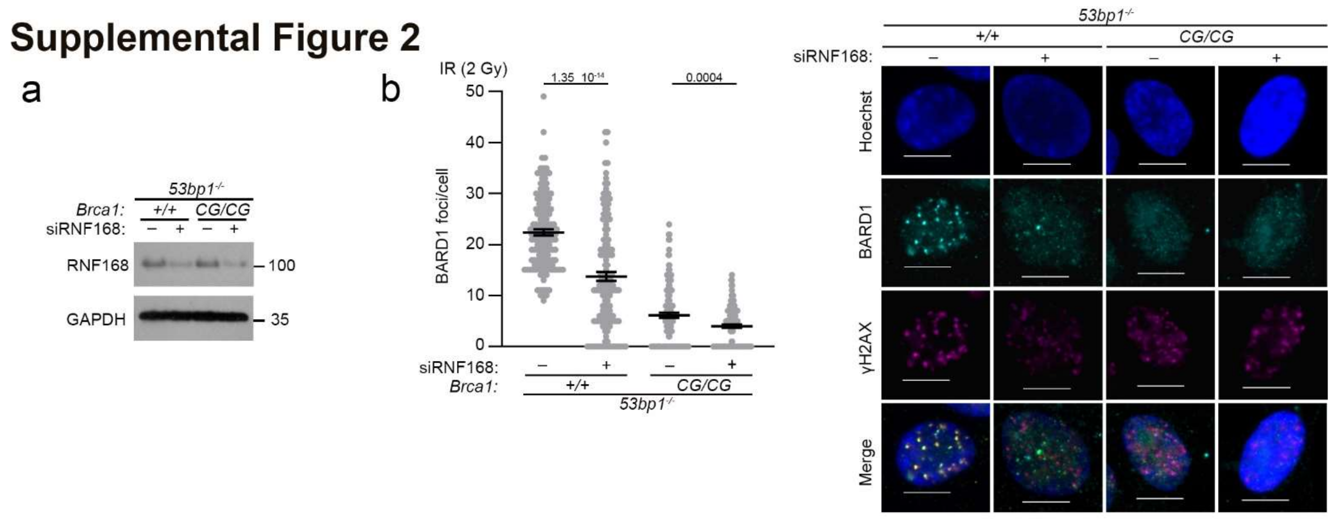

Supplementary Fig. 2. Impact of RNF168 depletion on BARD1 localisation.

a. Western blot analysis of RNF168 protein levels following non-targeting control (−) or RNF168 (+) targeting siRNA treatment.

b. Quantification of BARD1 foci 3 hours after 2 Gy IR exposure in EdU-positive MEFs of the genotypes shown and which were treated with NTC siRNA or RNF168 siRNA. n=150 cells from 3 biological replicates. Data are mean ± SEM. Images (right) show representative images of BARD1 foci in the genotypes shown with and without RNF168 depletion. The scale bar is 10 µm.

All statistical analysis in this figure was performed using a two-tailed Student's t-Test, without adjustment for multiple comparisons. Source data are provided as a Source Data file.

Supplemental Figure 3.

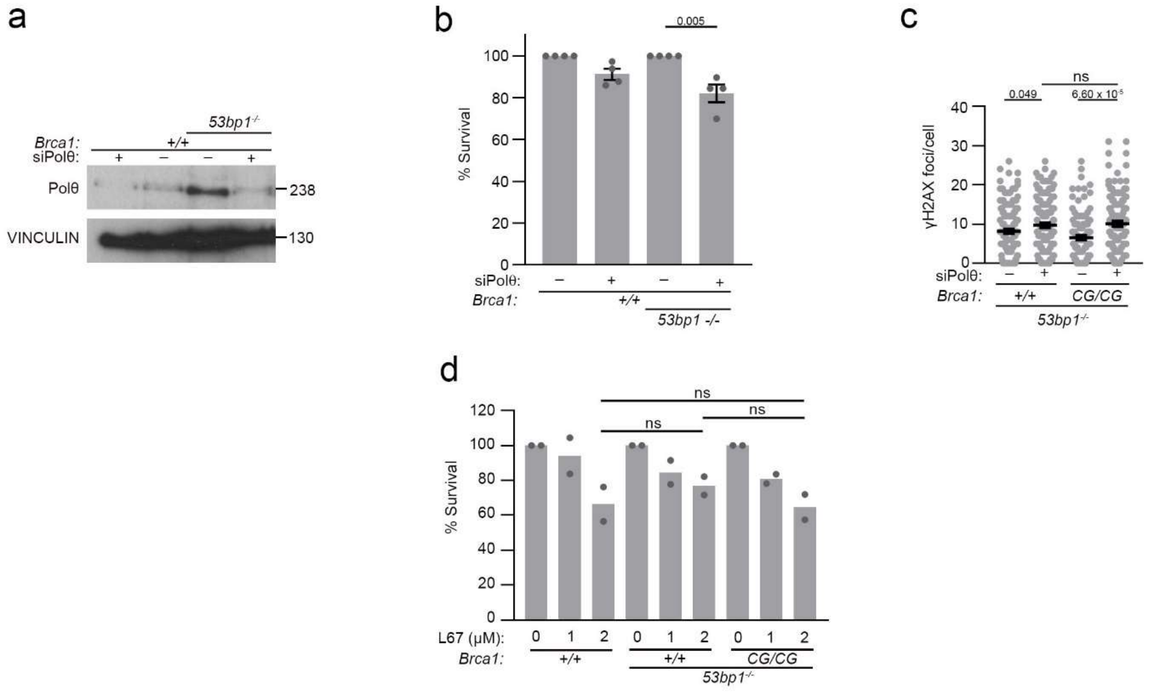

Supplementary Fig. 3. Assessment of Polθ siRNA and Ligase I/III inhibitor sensitivity.

a. Western blot of Polθ in genotypes shown, treated with control (−) siRNA or siRNA to Polθ (+).

b. Colony survival in MEFs of the genotypes shown treated with control (-) or Polθ (−) siRNA normalised to untreated genotype matched controls. n=4 biological repeats. Data are mean ± SEM.

c. γH2AX foci, in asynchronous cells of the genotypes shown (all are *53bp1-/-*), treated with non-targeting control (NTC) (−) or Polθ (+) siRNA. n=120 cells per condition from 3 biological replicates. Data are mean ± SEM.

d. Colony survival in MEFs of the genotypes shown treated with the Ligase I/III inhibitor, L67, normalised to untreated genotype matched controls. n=2 biological replicates. Data are mean ± SD.

All statistical analysis in this figure was performed using a two-tailed Student's t-Test, without adjustment for multiple comparisons. Source data are provided as a Source Data file.

Supplemental Figure 4.

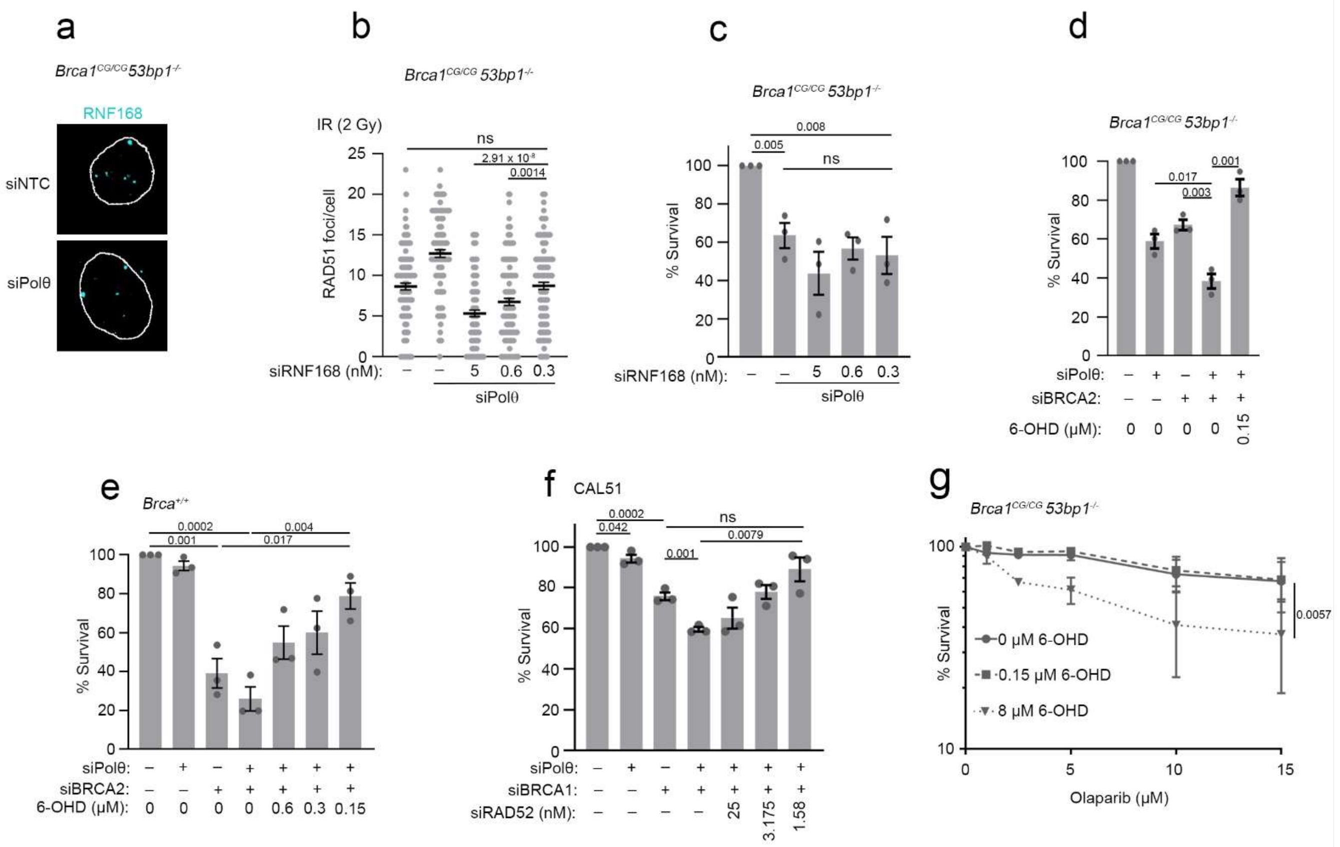

Supplementa Fig. 4. RNF168 depletion does not suppress the toxicity of Polθ depletion, low concentration RAD52 inhibition suppresses the toxicity of Polθ depletion in BRCA2 and BRCA1 depleted cells and has no impact on Olaparib sensitivity.

a. Representative images of RNF168 foci in *Brca1*$^{C61G/C61G}$ *53bp1*$^{-/-}$ cells, 72 hours after treatment with siNTC or siRNA targeting Polθ.

b. Quantification of RAD51 foci 4 hours post 2 Gy IR exposure, in EdU-positive *Brca1*$^{C61G/C61G}$ *53bp1*$^{-/-}$ cells treated with siRNA targeting Polθ (+), or control siRNA (−) with or without siRNA targeting RNF168. n=100 cells from 2 biological replicates. Data are mean ± SEM.

c. Colony survival in *Brca1*$^{C61G/C61G}$ *53bp1*$^{-/-}$ cells treated with siNTC or Polθ siRNA, with or without siRNA targeting RNF168. n=3 biological replicates, data are mean ± SEM.

d. Colony survival in *Brca1*$^{C61G/C61G}$ *53bp1*$^{-/-}$ cells treated with siNTC (−) or Polθ siRNA, with or without BRCA2 siRNA and the RAD52 inhibitor, 6-OHD. n=3 biological replicates, data are mean ± SEM.

e. Colony survival of wild-type MEFs treated with siNTC (−) or Polθ siRNA, with or without BRCA2 siRNA and the RAD52 inhibitor, 6-OHD. n=3 biological replicates, data are mean ± SEM.

f. Colony survival of CAL51 cells treated with BRCA1 siRNA and Polθ siRNA with decreasing amounts of RAD52 siRNA. n=3, data are mean ± SEM.

g. Colony survival of *Brca1*$^{C61G/C61G}$ *53bp1*$^{-/-}$ cells treated with the concentrations shown of RAD52 inhibitor 6-OHD and increasing doses of olaparib. n=2 biological replicates, data are mean ± SEM.

Ronson & Starowicz

Statistical analysis in b-f was performed using a two-tailed Student's t-Test, without adjustment for multiple comparisons. Statistical analysis in g was performed using a two-way ANOVA. Source data are provided as a Source Data file.

Supplemental Figure 5.

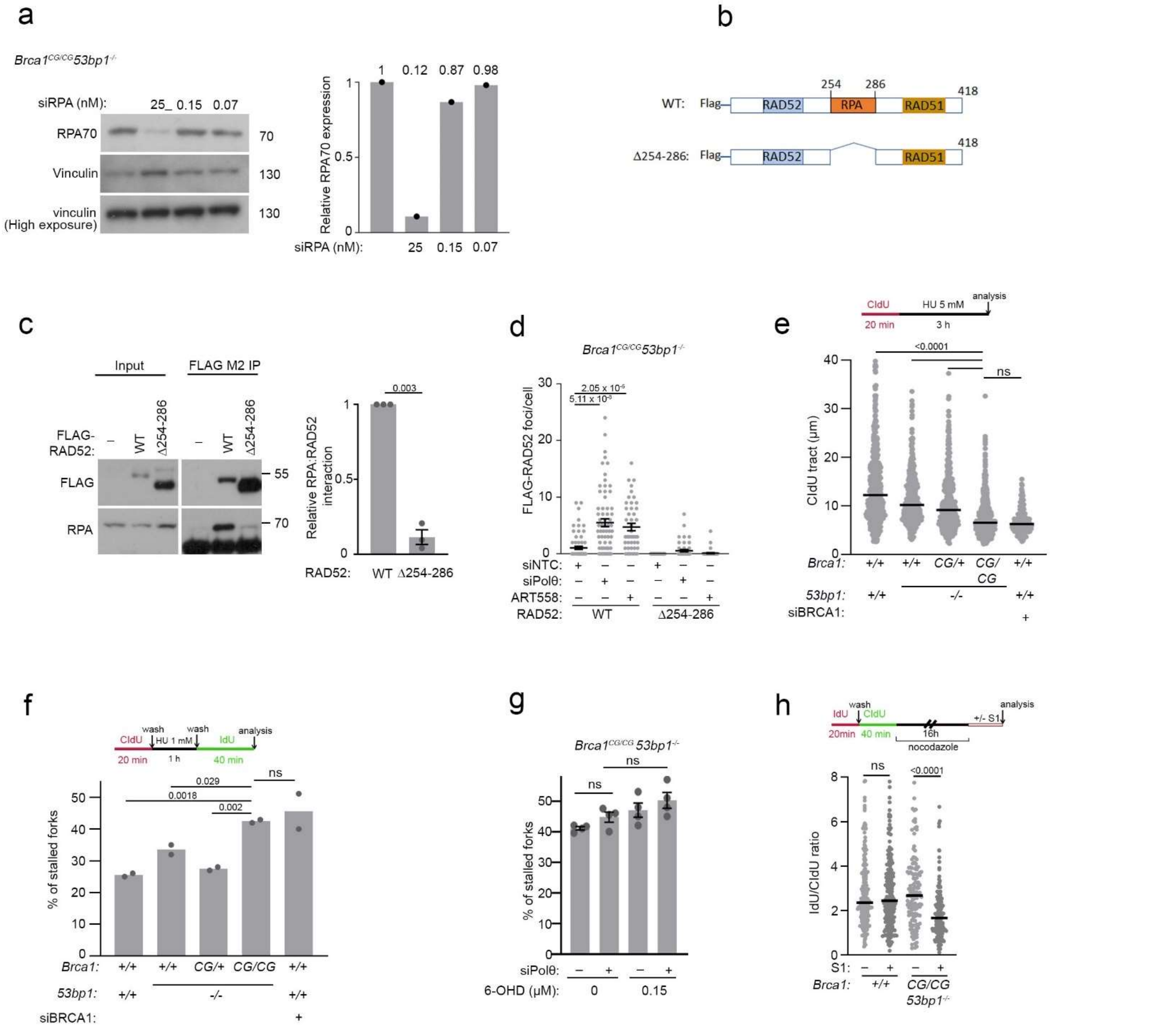

Supplementary Figure 5. Assessment of replication features for correlation with the Polθ:RAD52 relationship.

a. Western blot analysis of RPA70 protein levels following non-targeting control (−) or RPA70 targeting siRNA treatment with indicated concentrations.
b. Schematic of RAD52 constructs, illustrating the regions of RAD52 that interact with RAD51 and RPA.
c. Immunoprecipitation of FLAG-RAD52 mutants and immunoblot for FLAG-RAD52 and RPA. Graph (right) shows quantification from 3 independent experiments. Data are mean ± SEM.
d. FLAG-RAD52 foci generated by wild-type FLAG-RAD52 and FLAG-RAD52 missing amino acids 254–286 in asynchronous *Brca1*$^{C61G/C61G}$ *53bp1*$^{-/-}$ cells treated with siRNA to Polθ or with ART558 (10 µM). n≥45 cells per condition, from 2 biological replicates. Data are mean ± SEM.
e. Top, schematic of fork stability assay. CIdU tract length from cells with the illustrated genotypes treated with hydroxyurea (5 mM, 3 h). n>200 fibres from 3 biological replicates. Bars=median.
f. % fibres with first-label terminations from cells with the illustrated genotypes. n>800 fibres from 2 biological replicates. Data are mean ± SEM.
g. % fibres with first label terminations from *Brca1*$^{C61G/C61G}$ *53bp1*$^{-/-}$ cells treated with Polθ siRNA, and/or RAD52 inhibitor, 6-OHD. n>250 fibres from 4 biological replicates. Data are mean ± SEM.
h. S1 nuclease assay in G2/M in wild-type and *Brca1*$^{C61G/C61G}$ *53bp1*$^{-/-}$ cells, illustrated in the cartoon above, for ssDNA gaps formed in nascent DNA in the previous S phase. n>140 from 1 biological replicate. Bar=median.

Statistical analysis in c, d, f, and g was performed using a two-tailed Student's t-Test, without adjustment for multiple comparisons. Statistical analysis in e and h was performed using a Mann Whitney test. Source data are provided as a Source Data file.

We assessed stalled replication forks, the stability of which are reduced in *Brca1*$^{C61G/C61G}$ *53bp1*$^{-/-}$ cells (Supplemental Fig. 5e). While neither Polθ siRNA nor 0.15 µM 6-OHD, influenced stalled fork instability in HU-treated *Brca1*$^{C61G/C61G}$ *53bp1*$^{-/-}$ cells, their combination improved fork protection (Fig. 4h). These data suggest that while not affecting the amount of nascent DNA lost, Polθ depletion biases degradation towards a RAD52-mediated mechanism. Polθ and RAD52 also participate in the recovery of stalled forks[11,83,51,52,52,54] . We noted that the reduced restart of HU-stalled forks in Polθ depleted *Brca1*$^{C61G/C61G}$ *53bp1*$^{-/-}$ cells was worsened by RAD52 inhibitor addition (Supplemental Fig. 5f & g), consistent with a recent report of a synthetic reduction in fork restart[54.]. We next examined the exposure of the nucleotide analogue BrdU in cells labelled for 48 hours in non-denatured, native DNA fibres, indicating ssDNA. ssDNA lengths increased following treatment with Polθ siRNA and were MRE11-dependent, as previously reported[16,17] and were also suppressed by RAD52 inhibitor (Fig. 4i). However, since ssDNA lengths were increased after 0.15 µM 6-OHD RAD52 inhibitor treatment alone, which has no influence of cell survival (not shown), we conclude that gross ssDNA is unlikely to be the underlying structure for RAD52: Polθ synthetic lethality in *Brca1*$^{C61G/C61G}$ *53bp1*$^{-/-}$ cells. In summary, fork instability, poor fork restart and increased gross ssDNA do not correlate with the RAD52:Polθ viability relationship, suggesting they are unlikely to be reflective of where Polθ is most required.

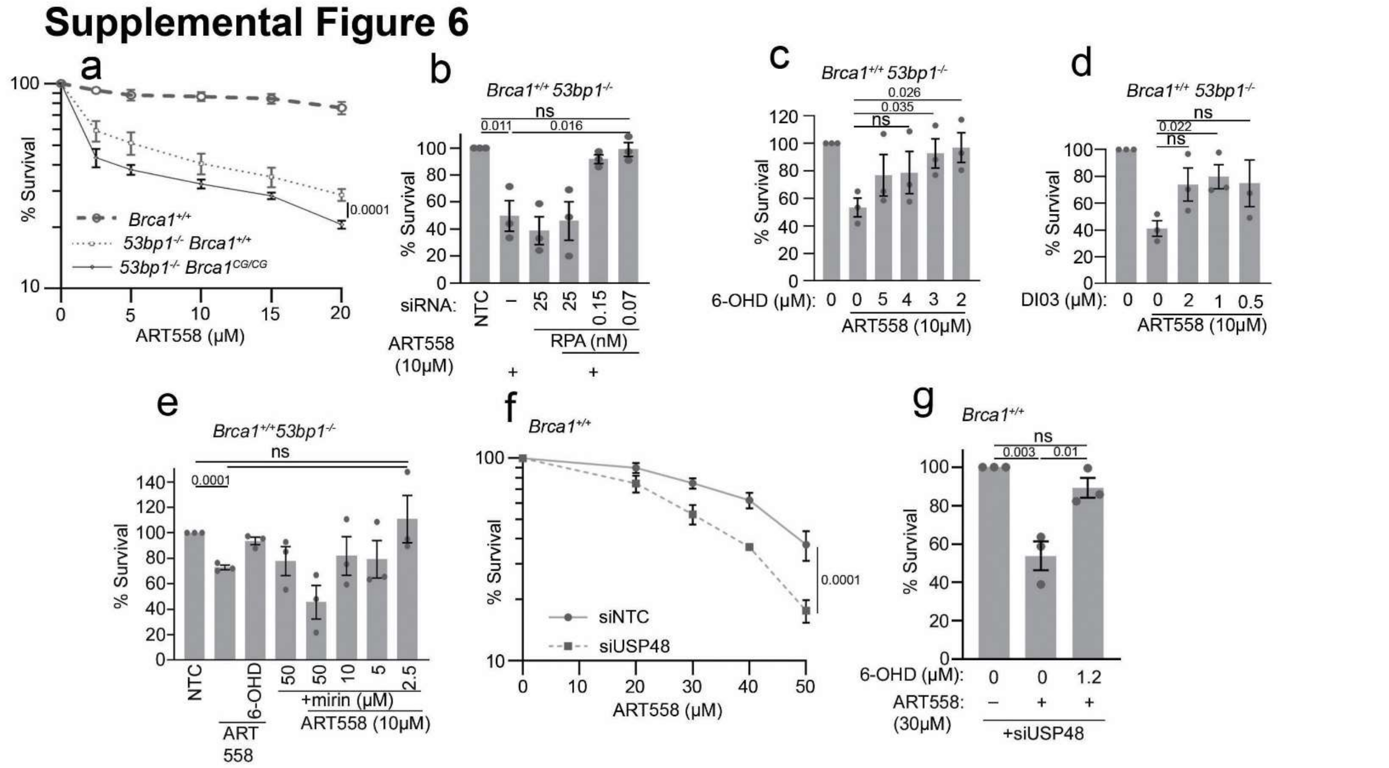

Supplementary Fig. 6. Synthetic lethality between 53BP1 loss and Polθ inhibition is suppressed by RAD52 inhibition.

a. Colony survival in wild-type (WT), *53bp1*$^{-/-}$ and *Brca1*$^{C61G/C61G}$ *53bp1*$^{-/-}$ cells treated with increasing doses of ART558 for 72 hours. n=6 biological repeats. Data are mean ± SEM.

b. Colony survival of *53bp1*$^{-/-}$ cells (with WT BRCA1), treated with 10 µM Polθ inhibitor ART558 and decreasing concentrations of RPA siRNA. n=4 biological repeats. Data are mean ± SEM.

c. Colony survival in *53bp1*$^{-/-}$ cells treated with 10 µM ART558 and decreasing doses of RAD52 inhibitor 6-OHD. n=3 biological repeats. Data are mean ± SEM.

d. Colony survival in *53bp1*$^{-/-}$ cells treated with 10 µM ART558 and decreasing doses of RAD52 inhibitor DI03. n=3 biological repeats. Data are mean ± SEM.

e. Colony survival of *53bp1*$^{-/-}$ cells, treated with 10 µM Polθ inhibitor ART558, 0.15 µM 6-OHD and the MRE11 inhibitor mirin. n=3 biological repeats. Data are mean ± SEM.

f. Colony survival of wild-type cells treated with non-targeting control (NTC) or USP48 siRNA and increasing concentrations of the Polθ inhibitor ART558. n=3 biological repeats. Data are mean ± SEM.

g. Colony survival of wild-type cells, treated with NTC siRNA (−) or USP48 siRNA and 30 µM Polθ inhibitor ART558 with either vehicle or 1.2 µM RAD52 inhibitor 6-OHD. n=3 biological repeats. Data are mean ± SEM.

Statistical analysis in b-e and g was performed using a two-tailed Student’s t-Test, without adjustment for multiple comparisons. Statistical analysis in a and f was performed using a two-way ANOVA. Source data are provided as a Source Data file.

Supplemental Figure 7

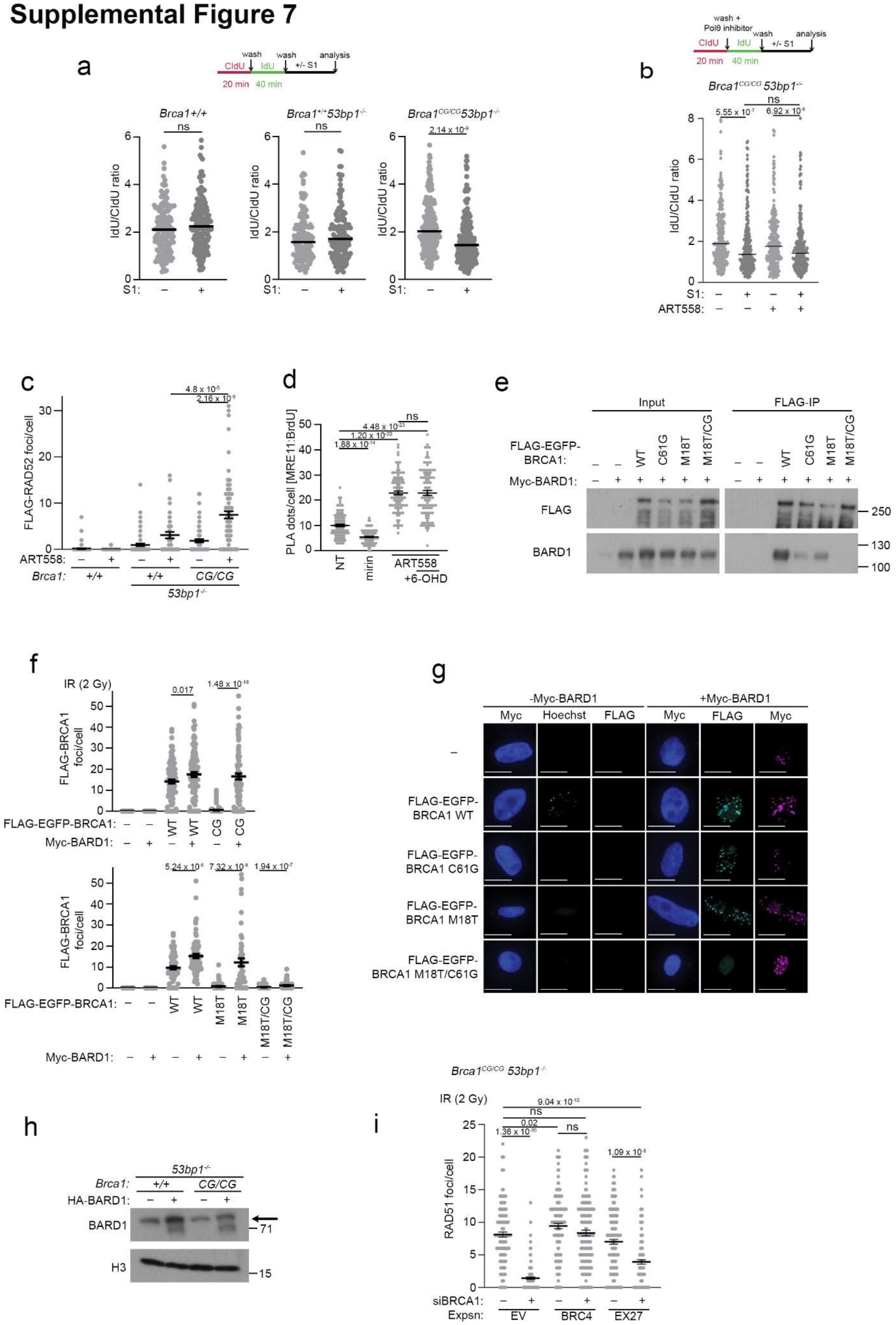

Supplementary Fig.7 BARD1 influence on C61G-BRCA1 foci formation and BRCA2 fragment RPA fusions on RAD51 foci formation in BRCA1-depleted cells.

a. S1 nuclease assay, illustrated in the cartoon above, for ssDNA gaps in nascent DNA in the genotypes shown. n>320 fibers per condition from 3 replicates. Bar=median.

b. S1 nuclease assay of *Brca1*$^{C61G/C61G}$ *53bp1*$^{-/-}$ cells with and without 10 µM ART558 treatment. n>220 fibres per condition from 3 replicates. Bar=median.

c. FLAG-RAD52 foci in asynchronous cells with the genotypes shown treated with Polθ inhibitor ART558.

d. Quantification of proximity-linked ligation assay foci (PLA) between MRE11 and BrdU. Cells were treated with the indicated inhibitors for 72 h and with 10 µM BrdU 48 h before fixation. n=100 cells from 2 biological replicates, bars depict mean ± SEM.

e. Representative blot of FLAG-immunoprecipitation of human FLAG-EGFP-tagged BRCA1 variants from U2OS cells expressing exogenous human myc-BARD1.

f. Quantification of FLAG-EGFP-tagged BRCA1 variant foci in Edu-positive cells with and without co-expression of human myc-BARD1 following a 2-hour recovery from 2 Gy IR. n=120 from 3 biological repeats (upper panel) and n=60 from 2 biological repeats (lower panel). Data are mean ± SEM.

g. Representative images of FLAG-EGFP-tagged BRCA1 foci and mc-tagged BARD1 foci from U2OS cells following a 2-hour recovery from 2 Gy IR. Scale bars represent 10 µm.

h. Expression levels of BARD1 in the MEF genotypes shown with (+) and without (−) co-expression of murine BARD1.

i. RAD51 foci in Edu-positive *Brca1*$^{C61G/C61G}$ *53bp1*$^{-/-}$ cells treated with non-targeting control (NTC), or BRCA1 siRNA and infected with empty pMSCV-IRES-EGFP (EV) retrovirus or those expressing RPA-70-BRCA2-BRC4 (BRC4) or RPA-70-BRCA2-Exon27 (Ex27). n=3 biological replicates, data are mean ± SEM.

All statistical analysis in this figure was performed using a two-tailed Student's t-Test, without adjustment for multiple comparisons. Source data are provided as a Source Data file.

Supplementary Table 1 – Antibodies.

Antibody (clone)	Host	Supplier	Cat. number	Lot number	Technique	Conc	RRID
β-actin	Rabbit	Abcam	Ab8227	GR3215935-1	WB	1:3000	AB_2305186
Human BARD1	Rabbit	Abcam	ab226854	GR3197067-8	WB	1:2000	N/A
Murine BARD1	Rabbit	Gift from R.Baer (McCarthy et al., 2003)	N/A	N/A	WB/IF	1:1000	N/A
Human BRCA1 (D-9)	Mouse	Santa Cruz	Sc6954	C2519	IF	1:500	AB_626761
Human BRCA1 (MS110)	Mouse	MERCK Millipore	OP94	3091924	WB	1:500	AB_213438
Murine BRCA1 (56E)	Rabbit	Gift from R.Baer	N/A	N/A	IF	1:1000	N/A
Murine BRCA1 (C40)*	Rabbit	Dundee cell Products	Custom design		IP		N/A
Murine BRCA1 (287.17)	Mouse	Santa Cruz	sc-135732	D2121	IF	1:50	AB_2243740
Murine BRCA1	Rabbit	Affinity Bioscience	AF6288	14f1430	WB	1:1000	AB_2835138
Polymerase theta	Rabbit	MyBiosource	MBS9612322	85i9616	WB	1:500	
CldU (BrdU)	Rat	Abcam	Ab6326	GR3173537-9	Fibres/IF	1:2000	AB_305426
Flag (M2)	Mouse	SigmaAldrich	F1804	SLBT7654	WB	1:1000	AB_262044
IdU (BrdU)	Mouse	BD Biosciences	347580	8151735	Fibres/PLA	1:500/ 1:200	AB_400326
γH2AX	Rabbit	Abcam	Ab2893	GR3242597-1	IF	1:2000	AB_303388
PALB2	Rabbit	Bethyl	A301.246A		WB	1:2000	AB_890607
RNF168	Sheep	Novus Biologicals	AF7217		IF/WB	1:1000	AB_10971653
RAD51 (Ab-1)	Rabbit	Calbiochem	PC130	3135376	PLA	1:100	AB_2238184
				3668125	IF	1:1000	
Mre11	Rabbit	Novus Biologicals	NB100-142	V-1	PLA	1:200	AB_10077796
RAD52	Sheep	Gift from Fena Ochs/Claudia Lukas	University of Copenhagen.		WB	1:1000	N/A
RPA70	Mouse	Abcam	Ab176467	GR3249141-1	WB	1:1000	N/A
Tubulin	Mouse	Santa Cruz	sc-5286	H0613	WB	1:5000	AB_628411
Vinculin [EPR8185]	Rabbit	Abcam	Ab129002	GR221671-50	WB	1:2000	AB_11144129
Donkey α Mouse AlexaFluor 488	Donkey	Life technologies	A21202	1975519	IF/fibres	1:5000	AB_141607
Donkey α Rabbit AlexaFluor 488	Donkey	Life technologies	A21206	1874771	IF	1:500	AB_2535792
Donkey α Mouse AlexaFluor 555	Donkey	Life technologies	A31570	1774719	IF/fibres	1:500	AB_2536180
Donkey α Rabbit AlexaFluor 555	Donkey	Life technologies	A31572	1945911	IF	1:500	AB_162543
Donkey α Rat AlexaFluor 555	Donkey	Life technologies	A21434	1987272	fibres	1:500	AB_2535855
Donkey α Rat AlexaFluor 488	Donkey	Life technologies	A21208	2480078	IF/fibres	1:500	AB_2535794
Rabbit α Mouse HRP	Rabbit	Dako	P0161	20062080	WB	1:10000	AB_2687969
Swine α Rabbit HRP	Swine	Dako	P0217	20047666	WB	1:10000	AB_2728719

****Antibody generation.*** Murine BRCA1 residues 1-300 bearing a Histidine tag and expressed in bacteria was used as an immunogen in Rabbits (Dundee cell products). The antibody is available on request to the corresponding authors subject to completion of a standard MTA.

Supplementary Table 2 - siRNA sequences

Target	siRNA Sequences	Supplier
NTC (Renilla	Sense: CUUACGCUGAGUACUUCGA[dT][dT]	Sigma
Luciferase)	Antisense: [Phos]UCGAAGUACUCAGCGUAA G[dT][dT]	Aldrich
mBRCA1	Sense: GGAUUUAUCUGCCGUCCAA [dT][dT]	Sigma
	Antisense: [Phos] UUGGACGGCAGAUAAAUCC[[dT][dT]	Aldrich
	Sense: GAACAGAGCAACUUGAAAC [dTdT]	
	Antisense: [Phos] AUUGUCUGUAUAGUCCACAGG [dT][dT]	
mRNF168	Sense: CCUUGGCUUCUCCUUUGAGUU [dT][dT]	Sigma
	Antisense: [Phos] AACUCAAAGGAGAAGCCAAGG [dT][dT]	Aldrich
mRAD52	Sense: UUGAAGGUCAUCGGGUAAUUA [dT][dT]	Sigma
	Antisense: [Phos]UAAUUACCCGAUGACCUUCAA [dT][dT]	Aldrich
	Sense: ACUAUCUGAGGUCACUAAAUA [dT][dT]	
	Antisense: [Phos] UAUUUAGUGACCUCAGAUAGU [dT][dT]	
	Sense: CCCACAUGACUCGAACAUUAA [dT][dT]	
	Antisense: [Phos] UUAAUGUUCGAGUCAUGUGGG [dT][dT]	
	Sense: CAGACUUAGAGGACAUCAUUA [dT][dT]	
	Antisense: [Phos] UAAUGAUGUCCUCUAAGUCUG [dT][dT]	
	Sense: GCAGAGACUCUGAACCUCAUA [dT][dT]	
	Antisense: [Phos] UAUGAGGUUCAGAGUCUCUGC [dT][dT]	
mPolθ	Sense: CCAGACUAAGAGUUCUCAUAA [dT][dT]	Sigma
	Antisense:[Phos] UUAUGAGAACUCUUAGUCUGG [dT][dT]	Aldrich
	Sense: CCAGGAAUCAAAGACGACAAU [dT][dT]	
	Antisense:[Phos] AUUGUCGUCUUUGAUUCCUGG [dT][dT]	
	Sense: CACGGAAGAAAGCGUUGUUUA [dT][dT]	
	Antisense:[Phos] UAAACAACGCUUUCUUCCGUG [dT][dT]	
mRADX	Sense: CAUAGAGGCCAGCCGUAUA [dT][dT]	Sigma
	Antisense:[Phos] UAUACGGCUGGCCUCUAUG [dT][dT]	Aldrich
	Sense: GAAAGUAUUCCACGGAAAUUU [dT][dT]	
	Antisense:[Phos] AAAUUUCCGUGGAAUACUUUC [dT][dT]	
	Sense: GGGAUAAUUACUGCUAUAAAG [dT][dT]	
	Antisense:[Phos] CUUUAUAGCAGUAAUUAUCCC [dT][dT]	
mBRCA2	Sense: CGGCGUUUCUAAAGAGUGCAU [dT][dT]	Sigma
	Antisense: [Phos] AUGCACUCUUUAGAAACGCCG [dT][dT]	Aldrich
mRPA32	Sense: AGUAAACCAGGACUGGAUUUC [dT][dT]	Sigma
	Antisense: [Phos] GAAAUCCAGUCCUGGUUUACU [dT][dT]	Aldrich
mRPA70	Sense: GCCCUGAAGAUCGCUAACAAA [dT][dT]	
	Antisense: [Phos] UUUGUUAGCGAUCUUCAGGGC [dT][dT]	
mUSP48	Sense: AUUCCUUUGUGGGCUUGACUA[dT][dT]	
	Antisense: [Phos] UAGUCAAGCCCACAAAGGAAU[dT][dT]	
	Sense: AUUCUGGCCACUACAUCGCAC[dT][dT]	
	Antisense: [Phos]GUGCGAUGUAGUGGCCAGAAU[dT][dT]	
hBRCA1	Sense: GCUCCUCUCACUCUUCAGU[dT][dT]	Sigma
	Antisense: [Phos] ACUGAAGAGUGAGAGGAGC [dT][dT]	Aldrich
	Sense: AAGCUCCUCUCACUCUUCAGU [dT][dT]	Sigma
	Antisense: [Phos]ACUGAAGAGUGAGAGGAGCUU [dT][dT]	Aldrich
hPolQ	Sense: CGUCGUCUCAUUCAAGUGUUA [dT][dT]	
	Antisense: [Phos]UAACACUUGAAUGAGACGACG [dT][dT]	
	Sense: CCUUAAGACUGUAGGUACU[dT][dT]	
	Antisense: [Phos]AGUACCUACAGUCUUAAGG [dT][dT]	
hRad52	Sense: CCACCAGAAACCACAAGCAAA [dT][dT]	Sigma
	Antisense: [Phos]UUUGCUUGUGGUUUCUGGUGG [dT][dT]	Aldrich
	Sense: CGGGUAAUUAAUCUGGCCAAU [dT][dT]	
	Antisense: [Phos]AUUGGCCAGAUUAAUUACCCG [dT][dT]	

Supplementary Table 3 – primer, template, and gRNA sequences

Gene	Point mutation (human)	Point mutation (murine)	Sequences (5'-3-)	Supplier
BARD1	L44R	L38R	Forward: GCTTGCCCGCCGGGAGAAGCTGCTG<br>Reverse: ACTGGGCATCCTGAGCCAACACAG	Sigma Aldrich
	F133A/D135A/A136E<br>(AAE)	F125A/D127A/A128E<br>(AAE)	Forward: CATTTTTATTGAATTCTTCTTCCTTTCTTCAGCACCAGCTAAA CTTGCCCTAGATG TGTTGTCTTTTGAAT<br>Reverse: ATTCAAAAGACAACACATCTAGGGCAAGTTTAGCTGGTGCT GAAGAAAGGAAGAAGAATTCAATAAAAATG	Sigma Aldrich
	A460T	A448T	Forward: CGGTGTCCATCCAGTATGGTCTTTAACATTTGGGT<br>Reverse: ACCCAAATGTTAAAGACCATACTGGATGGACACCG	Sigma Aldrich
	D712A	D700A	Forward: GATGGTCTGAGTCACAGCACTGTCTGGCTTGGG<br>Reverse: CCCAAGCCAGACAGTGCTGTGACTCAGACCATC	Sigma Aldrich
BRCA1	M18T	-	Forward: CAAAATGTCATTAATGCTTTGCAGAAAATCTTAGAGTG	Sigma Aldrich
Rosa 26 F			AGAAAACTGGCCCTTGCCATT	Sigma Aldrich
Roas26 R			CAGCCTCGATTTGTGGTGTATG	Sigma Aldrich
Rosa26 HR assay template			GGGGGAGTCGTTTTACCCGCCGCCGGCCGGGCCTCGTCGTCTGATTGGC TCTCGGGGCCCAGAAAACTGGCCCTT GCCATTGGCTCGTGTTCGTGCAA GTTGAGTCCATCCGCCGGCCAGCGGGGGCGGCGAGGAGGCGCTCCCAG GTTC CGGCCCTCCCCTCGGCCCCGCGCCGCAGAGTCTGGCCGCGCGCCC CTGCGCAACGTGGCAGGAAGCGCGCGCTG GGGGCGGGGACGGGCAGT AGGGCTGAGCGGCTGCGGGGCGGGTGCAAGCACGTTTCCGACTTGAGT TGCCTCAA GAGGGGCGTGCTGAGCCAGACCTCCATCGCGCACTCCGGG GAGTGGAGGGAAGGAGCGAGGGCTCAGTTGGGCT GTTTTGGAGGCAG GAAGCACTTGCTCTCCCAAAGTCGCTCTGAGTTGTTATCAGTAAGGGAGC TGCAGTGGAGTAG GCGGGGAGAAGGCCGCACCCTTCTCCGGAGGGGG GAGGGGAGTGTTGCAATACCTTTCTGGGAGTTCTCTGCTGC CTCCTGGC TTCTGAGGACCGCCCTGGGCCTGGGAGAATCCCTTCCCCCTCTTCCCTCG TGATCTGCAACTCCAGTCTTTCTAGAAGtactGCGGGAGTCTTCTGGGCA GGCTTAAAGGCTAACCTGGTGTGTGGGCGTTGTCCTGCAGGGG AATTG AACAGGTGTAAAATTGGAGGGACAAGACTTCCCACAGATTTTCGGTTTT GTCGGGAAGTTTTTTAATAGGGG CAAATAAGGAAAATGGGAGGATAG GTAGTCATCTGGGGTTTTATGCAGCAAAACTACAGGTTATTATTGCTTGT GAT CCGCCTCGGAGTATTTTCCATCGAGGTAGATTAAAGACATGCTCAC CCGAGTTTTATACTCTCCTGCTTGAGATCC TTACTACAGTATGAAATTAC AGTGTCGCGAGTTAGACTATGTAAGCAGAATTTTAATCATTTTTAAAGA GCCCAGTAC TTCATATCCATTTCTCCCGCTCCTTCTGCAGCCTTATCAAAA GGTATTTTAGAACACTCATTTTAGCCCCATTTTCATT TATTATACTGGCTT ATCCAACCCCTAGACAGAGCATTGGCATTTTCCCTTTCCTGATCTTAGAA GTCTGATGACTCATGAAACCAGACA	Genscript
Rosa26 gRNA			ACTCCAGTCTTTCTAGAAGA	

Supplementary Table 4 - Details of chemical reagents, DNA damaging agents and inhibitors

Inhibitor	Company	Catalogue Number	Target	Concentration
**Olaparib**	Selleckchem	S1060	PARP	1-20 µM
**BrdU**	Sigma-Aldrich	B5002	Thymidine analogue	5-100 nM
**Cisplatin**	Sigma-Aldrich	PHR1624	DNA-crosslinker	1-5 µM
**CldU**	Sigma-Aldrich	C6891	Thymidine analogue	250 µM
**EdU**	Thermofisher	A10044	Thymidine analogue	10 µM
**IdU**	Sigma-Aldrich	I7125	Thymidine analogue	25 µM
**Hydroxyurea**	Sigma-Aldrich	H8627	Ribonucleoside reductase	1-10 mM
**Colcemid**	Gibco	15212012	Microtubule poison	0.05 µg/ml
**6-OH-DL-DOPA**	Sigma	H2380	RAD52/APE1	0.15-5 µM
**DI03**	Sigma	SML2496	RAD52	0.5-10 µM
**Novobiocin**	Selleckchem	NSC2382	Polymerase Theta	50-200 µM
**ART558**	Artios	n/a	Polymerase Theta	2.5-20 µM
**L67**	Sigma-Aldrich	SML1797	DNA Ligase I/III	1-2 µM
