# Supplementary material for: Mechanisms of synthetic lethality between BRCA1/2 and 53BP1 deficiencies and DNA polymerase theta targeting
Source: Nat Commun. 2023 Nov 29;14:7834. doi: 10.1038/s41467-023-43677-2 (PMC10687250; doi:10.1038/s41467-023-43677-2)
Supplement: Supplementary file 3 — Reporting Summary [file 41467_2023_43677_MOESM3_ESM.pdf]

nature portfolio

Corresponding author(s): JR Morris

Last updated by author(s): 01/11/2023

Reporting Summary

Nature Portfolio wishes to improve the reproducibility of the work that we publish. This form provides structure for consistency and transparency in reporting. For further information on Nature Portfolio policies, see our Editorial Policies and the Editorial Policy Checklist.

Statistics

For all statistical analyses, confirm that the following items are present in the figure legend, table legend, main text, or Methods section.

n/a	Confirmed	
☐	☒	The exact sample size (*n*) for each experimental group/condition, given as a discrete number and unit of measurement
☐	☒	A statement on whether measurements were taken from distinct samples or whether the same sample was measured repeatedly
☐	☒	The statistical test(s) used AND whether they are one- or two-sided<br>*Only common tests should be described solely by name; describe more complex techniques in the Methods section.*
☒	☐	A description of all covariates tested
☒	☐	A description of any assumptions or corrections, such as tests of normality and adjustment for multiple comparisons
☐	☒	A full description of the statistical parameters including central tendency (e.g. means) or other basic estimates (e.g. regression coefficient) AND variation (e.g. standard deviation) or associated estimates of uncertainty (e.g. confidence intervals)
☐	☒	For null hypothesis testing, the test statistic (e.g. *F*, *t*, *r*) with confidence intervals, effect sizes, degrees of freedom and *P* value noted<br>*Give P values as exact values whenever suitable.*
☒	☐	For Bayesian analysis, information on the choice of priors and Markov chain Monte Carlo settings
☒	☐	For hierarchical and complex designs, identification of the appropriate level for tests and full reporting of outcomes
☒	☐	Estimates of effect sizes (e.g. Cohen's *d*, Pearson's *r*), indicating how they were calculated

Our web collection on statistics for biologists contains articles on many of the points above.

Software and code

Policy information about availability of computer code

Data collection	Data collection was performed using the following software; Microscopy Images using LasX (Version 3.3.0.16799)
Data analysis	Data analysis was performed using Graphpad Prism (Version 7.03), Microsoft Excel 365 (Version 2310), and ImageJ (Version 1.47). DNA sequencing data was analysed using Guppy 5, Canu (2.2), Minimap2 (v2.24), CRISPResso2 (v2.2.7), and MEDJED (v1.2.0)

For manuscripts utilizing custom algorithms or software that are central to the research but not yet described in published literature, software must be made available to editors and reviewers. We strongly encourage code deposition in a community repository (e.g. GitHub). See the Nature Portfolio guidelines for submitting code & software for further information.

Data

Policy information about availability of data

All manuscripts must include a data availability statement. This statement should provide the following information, where applicable:

- Accession codes, unique identifiers, or web links for publicly available datasets
- A description of any restrictions on data availability
- For clinical datasets or third party data, please ensure that the statement adheres to our policy

All data generated in the study are included in this published article, including supplimentary figures, or are available from the authors upon reasonable request. Source data for this manuscript is available here: https://doi.org/10.6084/m9.figshare.24270799. Sequencing data were aligned using reference genome mm10 (GCF_000001635.20).

Research involving human participants, their data, or biological material

Policy information about studies with human participants or human data. See also policy information about sex, gender (identity/presentation), and sexual orientation and race, ethnicity and racism.

Reporting on sex and gender	Not applicable
Reporting on race, ethnicity, or other socially relevant groupings	Not applicable
Population characteristics	Not applicable
Recruitment	Not applicable
Ethics oversight	Not applicable

Note that full information on the approval of the study protocol must also be provided in the manuscript.

Field-specific reporting

Please select the one below that is the best fit for your research. If you are not sure, read the appropriate sections before making your selection.

☒ Life sciences ☐ Behavioural & social sciences ☐ Ecological, evolutionary & environmental sciences

For a reference copy of the document with all sections, see nature.com/documents/nr-reporting-summary-flat.pdf

Life sciences study design

All studies must disclose on these points even when the disclosure is negative.

Sample size	Sample sizes were based on preliminary data and published studies, to identify statistically significant changes in each relevant assay.
Data exclusions	No data was excluded from the study.
Replication	All experiments were independently replicated and this is reported for each experiment in the corresponding figure legend.
Randomization	Randomization was not relevant to this study, as cell lines with defined genetic backgrounds form the basis of the work.
Blinding	Blinding was not used in this study - in many cases, due to the experimental setup, blinding of samples was not possible.

Reporting for specific materials, systems and methods

We require information from authors about some types of materials, experimental systems and methods used in many studies. Here, indicate whether each material, system or method listed is relevant to your study. If you are not sure if a list item applies to your research, read the appropriate section before selecting a response.

Materials & experimental systems

n/a	Involved in the study	
☐	☒	Antibodies
☐	☒	Eukaryotic cell lines
☒	☐	Palaeontology and archaeology
☒	☐	Animals and other organisms
☒	☐	Clinical data
☒	☐	Dual use research of concern
☒	☐	Plants

Methods

n/a	Involved in the study	
☒	☐	ChIP-seq
☒	☐	Flow cytometry
☒	☐	MRI-based neuroimaging

Antibodies

Antibodies used	β-actin Rabbit Abcam Ab8227 GR3215935-1<br>Human BARD1 Rabbit Abcam ab226854 GR3197067-8<br>Murine BARD1 Rabbit Gift from R.Baer (McCarthy et al., 2003)<br>Human BRCA1 (D-9) Mouse Santa Cruz Sc6954 C2519

Human BRCA1 (MS110) Mouse MERCK Millipore OP94 3091924
Murine BRCA1 (56E) Rabbit Gift from R.Baer
Murine BRCA1 (C40)* Rabbit Dundee cell Products Custom design
Murine BRCA1 (287.17) Mouse Santa Cruz sc-135732 D2121
Murine BRCA1 Rabbit Affinity Bioscience AF6288 14f1430
Polymerase theta Rabbit MyBiosource MBS9612322 85i9616
CldU (BrdU) Rat Abcam Ab6326 GR3173537-9
Flag (M2) Mouse SigmaAldrich F1804 SLBT7654
IdU (BrdU) Mouse BD Biosciences 347580 8151735
γH2AX Rabbit Abcam Ab2893 GR3242597-1
PALB2 Rabbit Bethyl A301.246A
RNF168 Sheep Novus Biologicals AF7217
RAD51 (Ab-1) Rabbit Calbiochem PC130 3135376 3668125
Mre11 Rabbit Novus Biologicals NB100-142 V-1
RAD52 Sheep Gift from Fena Ochs/Claudia Lukas University of Copenhagen.
RPA70 Mouse Abcam Ab176467 GR3249141-1
Tubulin Mouse Santa Cruz sc-5286 H0613
Vinculin [EPR8185] Rabbit Abcam Ab129002 GR221671-50
Donkey α Mouse AlexaFluor 488 Donkey Life technologies A21202 1975519
Donkey α Rabbit AlexaFluor 488 Donkey Life technologies A21206 1874771
Donkey α Mouse AlexaFluor 555 Donkey Life technologies A31570 1774719
Donkey α Rabbit AlexaFluor 555 Donkey Life technologies A31572 1945911
Donkey α Rat AlexaFluor 555 Donkey Life technologies A21434 1987272
Donkey α Rat AlexaFluor 488 Life technologies A21208 2480078
Rabbit α Mouse HRP Rabbit Dako P0161 20062080
Swine α Rabbit HRP Swine Dako P0217 20047666

Validation

β-actin Rabbit (https://www.abcam.com/products/primary-antibodies/beta-actin-antibody-ab8227.html)
Human BARD1 (https://www.abcam.com/products/primary-antibodies/bard1-antibody-ab226854.html)
Murine BARD1 (McCarthy et al., 2003)
Human BRCA1 (D-9) (https://www.scbt.com/p/brca1-antibody-d-9)
Human BRCA1 (MS110) (https://www.merckmillipore.com/GB/en/product/Anti-BRCA1-Ab-1-Mouse-mAb-MS110,EMD_BIO-OP92)
Murine BRCA1 (56E) Rabbit Gift from R.Baer
Murine BRCA1 (C40)* Rabbit Dundee cell Products Custom designA
Murine BRCA1 (287.17) (https://www.scbt.com/p/brca1-antibody-287-17)
Murine BRCA1 (https://www.affbiotech.com/goods-1886-AF6288-BRCA1_Antibody.html)
Polymerase theta (https://www.mybiosource.com/polyclonal-polq-human-mouse-rat-antibody/dna-polymerase-theta/9612322)
CldU (BrdU) (https://www.abcam.com/products/primary-antibodies/brdu-antibody-bu175-icr1-proliferation-marker-ab6326.html)
Flag (M2) (https://www.sigmaaldrich.com/GB/en/product/sigma/f1804)
IdU (BrdU) (https://www.bdbiosciences.com/en-gb/products/reagents/flow-cytometry-reagents/clinical-discovery-research/single-color-antibodies-ruo-gmp/purified-mouse-anti-brdu.347580)
γH2AX (https://www.abcam.com/products/primary-antibodies/gamma-h2ax-phospho-s139-antibody-ab2893.html)
PALB2 (https://www.fortislife.com/products/primary-antibodies/rabbit-anti-palb2-antibody/BETHYL-A301-246)
RNF168 (https://www.novusbio.com/products/rnf168-antibody_af7217)
RAD51 (Ab-1) (https://www.merckmillipore.com/GB/en/product/Anti-Rad51-Ab-1-Rabbit-pAb,EMD_BIO-PC130)
Mre11 (https://www.novusbio.com/products/mre11-antibody_nb100-142)
RAD52 Gift from Fena Ochs/Claudia Lukas
RPA70 (https://www.abcam.com/products/primary-antibodies/rpa70-antibody-8c3-d12-h10-ab176467.html)
Tubulin (https://www.scbt.com/p/alpha-tubulin-antibody-b-7)
Vinculin [EPR8185] (https://www.abcam.com/products/primary-antibodies/vinculin-antibody-epr8185-ab129002.html)

The specificity of BRCA1 antibody from Dundee Biosciences was confirmed by Western blot comparing cells treated with non-targetting and BRCA1-targetting siRNA.

Eukaryotic cell lines

Policy information about cell lines and Sex and Gender in Research

Cell line source(s)

FlpIn U2OS and FlpIn HEK293T cell lines were from Morris lab stocks. To generate MEF lines, Brca1C61G/ + 53bp1-/- male and female animals were mated to generate littermates of required Brca1 genotypes. Pregnant mice were euthanised 13.5 days after mating and the embryos were dissected into media to allow fibroblasts to grow out. MEFs were immortalised by transduction with the SV40 large T antigen (pBsSVD2005, AdGene) using FuGENE (Promega) This was done in accordance with the UK Animals (Scientific Procedures) Act 1986 Home Office regulations under the authority of PPL70/8013.

Authentication

None of the cell lines used were authenticated

Mycoplasma contamination

All cell lines tested negative for mycoplasma contamination.

Commonly misidentified lines
(See ICLAC register)

No commonly misidentified cell lines were used.

Plants

Seed stocks	*Report on the source of all seed stocks or other plant material used. If applicable, state the seed stock centre and catalogue number. If plant specimens were collected from the field, describe the collection location, date and sampling procedures.*
Novel plant genotypes	*Describe the methods by which all novel plant genotypes were produced. This includes those generated by transgenic approaches, gene editing, chemical/radiation-based mutagenesis and hybridization. For transgenic lines, describe the transformation method, the number of independent lines analyzed and the generation upon which experiments were performed. For gene-edited lines, describe the editor used, the endogenous sequence targeted for editing, the targeting guide RNA sequence (if applicable) and how the editor was applied.*
Authentication	*Describe any authentication procedures for each seed stock used or novel genotype generated. Describe any experiments used to assess the effect of a mutation and, where applicable, how potential secondary effects (e.g. second site T-DNA insertions, mosiacism, off-target gene editing) were examined.*
